# Association between atherogenic index of plasma and stroke risk: A cross-sectional analysis of NHANES data

**DOI:** 10.1097/MD.0000000000047297

**Published:** 2026-01-23

**Authors:** Ziyi Liang, Huasheng Zhang, Yang Xiong, Xiaohu Zhang, Yuliang Chen, Peilong Li, Qiyou Yi, Kangkang Xia, Jun Liang

**Affiliations:** a Department of Neurosurgery, The Affiliated Hospital of Xuzhou Medical University, Xuzhou, Jiangsu Province, China.

**Keywords:** cross-sectional study, NHANES, plasma atherosclerosis index, population-based study, stroke

## Abstract

While metabolic and inflammatory factors have long been associated with stroke, emerging lipid-based markers such as the atherogenic index of plasma (AIP) may offer additional predictive value. This study aimed to evaluate the association between AIP and stroke incidence using a nationally representative dataset. A cross-sectional analysis was performed using data from the 2005 to 2018 National Health and Nutrition Examination Survey (NHANES), focusing on adults with complete records for AIP and stroke diagnosis. AIP was defined as the base-10 logarithm of the triglyceride-to-high-density lipoprotein cholesterol cholesterol ratio, measured in mmol/L. We applied weighted multivariable logistic regression models and generalized additive models to assess linear and nonlinear associations between AIP and stroke prevalence. Threshold effects were explored via 2-piecewise linear regression. We further examined interactions across subgroups to test for effect modification. Among 16,834 eligible participants, 510 (3.03%) had a history of stroke. Stroke prevalence increased progressively across ascending AIP tertiles (*Q*_1_: 39.05%, *Q*_2_: 44.95%, *Q*_3_: 53.45%; *P* < .0001). Adjusted analyses revealed a statistically significant positive association between AIP and stroke. Specifically, each 1-unit increase in AIP was associated with a 38% increase in stroke risk (model III: odds ratio = 1.38; 95% confidence interval: 1.04–1.83). No significant interactions were observed across major demographic or clinical subgroups (*P* for interaction > .05). Higher AIP values are independently associated with increased odds of stroke. These findings suggest that AIP could serve as a useful marker in evaluating cerebrovascular risk profiles in the general population.

## 1. Introduction

Stroke remains a major public health challenge worldwide, with an estimated 15 million new cases reported each year.^[1]^ Despite improvements in prevention and treatment, it continues to be a leading cause of mortality and long-term disability.^[2]^ As populations age, the societal and economic burdens of stroke are expected to intensify,^[3,4]^ underscoring the need to identify modifiable risk factors that can support early prevention strategies.

Lipid metabolism plays a crucial role in vascular health, and recent attention has shifted toward more nuanced lipid-related indices that may better capture cardiovascular risk. One such marker is the atherogenic index of plasma (AIP), defined as the logarithm of the ratio of triglycerides to high-density lipoprotein cholesterol (log[TG/HDL-C]).^[5]^ Introduced by Dobiásová and Frohlich, AIP serves as an integrated indicator of pro-atherogenic and anti-atherogenic lipid profiles.^[6]^ Previous studies have associated elevated AIP with increased risk for conditions such as coronary artery disease,^[7]^ peripheral artery disease,^[5]^ and metabolic syndrome (AIP identifies subjects with severe liver steatosis). These findings suggest that AIP may also have relevance in cerebrovascular disease risk assessment.

However, no large-scale epidemiological studies have systematically examined the association between AIP and stroke. Using data from the US National Health and Nutrition Examination Survey (NHANES), this study aims to explore the potential relationship between AIP levels and stroke prevalence in a representative adult population.

## 2. Materials and methods

This cross-sectional study utilized publicly available data from the NHANES, an ongoing program initiated in the early 1960s to monitor the health and nutritional status of the civilian, non-institutionalized US population. NHANES employs a multistage, stratified, and probability-clustered sampling strategy to ensure national representativeness. All procedures are approved by the National Center for Health Statistics (NCHS) Research Ethics Review Board, and written informed consent was obtained from all participants. Additional details about the study design and protocols can be accessed at https://www.cdc.gov/nchs/nhanes/.

**Figure 1.**
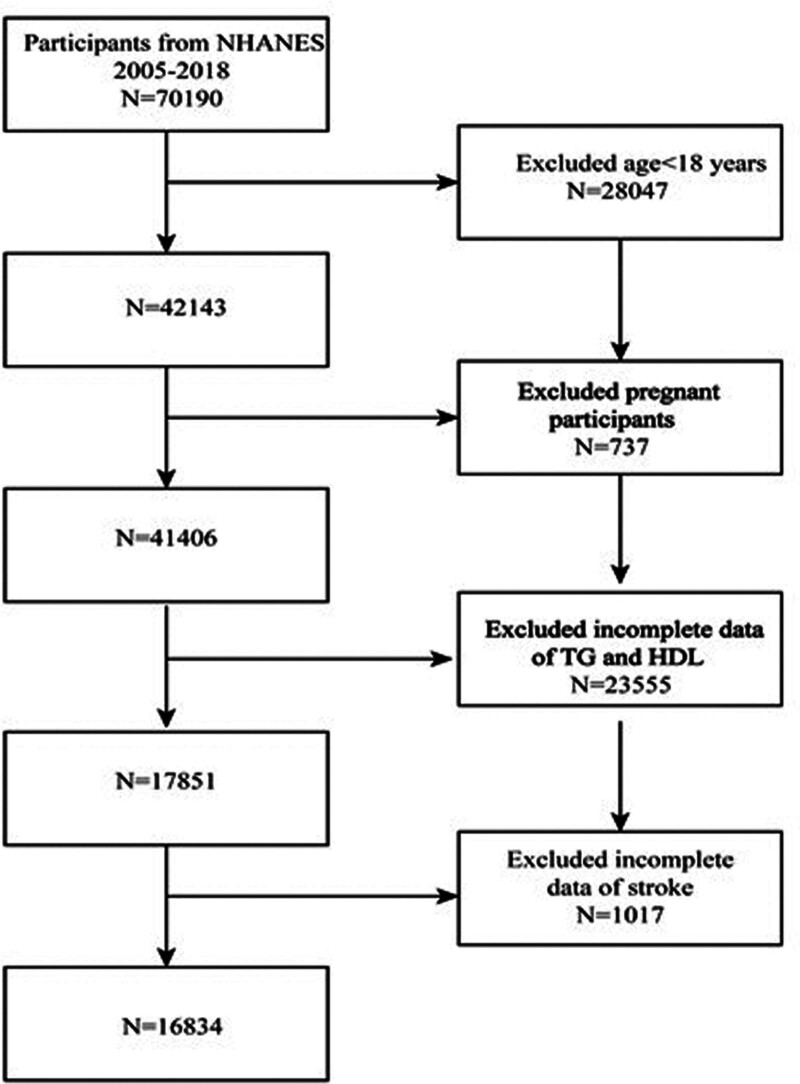
Study population selection flowchart. Flow diagram showing the selection process for study participants from the National Health and Nutrition Examination Survey (NHANES) 2005–2018 cycles. Starting from 70,190 initial participants, exclusions were applied sequentially: participants under 18 years of age, pregnant women, and those with missing data for atherogenic index of plasma calculation (serum triglycerides or high-density lipoprotein cholesterol) or stroke history. The final analytic sample comprised 16,834 participants who met all inclusion criteria and had complete data for the primary analysis. HDL = high-density lipoprotein, NHANES = National Health and Nutrition Examination Survey, TG = triglycerides.

For the present analysis, data from 7 continuous NHANES cycles (2005–2018) were pooled. We included individuals aged 18 years or older who had complete data available on serum TG, HDL-C, and stroke history. Participants were excluded if they were under 18 years of age, currently pregnant, or missing relevant data for AIP calculation or stroke status. Of the initial 70,190 participants, a total of 16,834 met the eligibility criteria and were included in the final analysis (see Fig. 1 for the inclusion flowchart).

### 2.1. Ethical considerations

This research is a retrospective analysis of publicly available and fully de-identified data from the NHANES database. The original NHANES studies were approved by the National Center for Health Statistics Research Ethics Review Board, and all participants provided written informed consent. The dataset was accessed in December 2023. The authors had no access to personally identifiable information at any stage of the study. Therefore, no additional ethical approval was required according to federal regulations.

### 2.2. Assessment of the plasma atherosclerosis index

The AIP is recognized as a robust biomarker for evaluating atherosclerosis and cardiovascular disease risk. It is calculated as the base-10 logarithm of the ratio of TG to HDL-C, with both parameters measured in mmol/L. Blood samples from NHANES participants were obtained and analyzed according to standardized protocols established by the Centers for Disease Control and Prevention. HDL-C levels were determined using either direct immunoassay or precipitation methods.^[8]^ In this study, AIP was analyzed as a continuous variable. Additionally, participants were stratified into tertiles based on AIP values to facilitate subgroup analysis. AIP was regarded as the primary exposure variable in our investigation.

### 2.3. Definition of stroke

Stroke was defined based on participants’ responses to a structured medical history questionnaire. Individuals were considered to have experienced a stroke if they answered “yes” to the question: “Has a doctor or other health professional ever told you that you had a stroke?” It is important to acknowledge that self-reported diagnoses are subject to recall bias, which may influence the accuracy of data interpretation.^[9]^

### 2.4. Covariates

Guided by previous literature and biological plausibility, we included a comprehensive set of covariates that may confound the association between AIP and stroke. Demographic characteristics – including age, sex, race/ethnicity, education level, marital status, family income, smoking status, and alcohol consumption – were collected using standardized questionnaires and face-to-face interviews. Physical examinations and laboratory tests were conducted by trained healthcare professionals at Mobile Examination Centers.

Race/ethnicity was categorized into 5 groups: non-Hispanic White, non-Hispanic Black, other Hispanic, Mexican American, and other races. Education level was stratified as less than high school, high school graduate, and above high school. Smoking status was defined as having smoked more than 100 cigarettes in a lifetime, regardless of current smoking behavior.^[10]^ Participants who had consumed at least 12 alcoholic beverages in the past year were considered drinkers.^[11]^

Body mass index (BMI) was calculated as weight (kg) divided by height squared (m²) and was used to categorize individuals as overweight (BMI > 25) or obese (BMI > 30) based on established criteria.^[12]^ Hypertension was defined by meeting any of the following: average systolic blood pressure ≥ 140 mm Hg, diastolic blood pressure ≥ 90 mm Hg, a prior diagnosis of hypertension by a healthcare professional, or current use of antihypertensive medication.^[13]^

Other covariates included laboratory-measured parameters such as low-density lipoprotein cholesterol (LDL-C), total cholesterol (TC), alanine aminotransferase (ALT), aspartate aminotransferase (AST), hemoglobin (HGB) concentration, red blood cell (RBC) count, platelet (PLT) count, and monocyte count – all obtained through standardized testing procedures.

### 2.5. Statistical analysis

Given the complex, multistage sampling design of the NHANES survey, all analyses incorporated appropriate sampling weights corresponding to each survey cycle to ensure nationally representative estimates of health-related statistics.^[14-16]^ Data distribution was assessed prior to statistical analysis. For variables that did not meet normality assumptions, appropriate statistical methods were selected, with results presented accordingly. Continuous variables were reported as weighted means with standard deviations, while categorical variables were expressed as weighted frequencies and percentages. Group differences across AIP tertiles were assessed using the weighted Student *t* test for continuous variables and the weighted chi-square test for categorical variables.

To evaluate the association between AIP and stroke, we performed both univariate and multivariate logistic regression analyses. Multivariate logistic regression models were constructed in 3 stages: model 1 included no covariate adjustments; model 2 adjusted for demographic factors including sex, age, race/ethnicity, education level, marital status, and family income-to-poverty ratio; and model 3 further adjusted for clinical and biochemical variables, including LDL-C, TC, ALT, AST, HGB concentration, RBC count, PLT count, monocyte count, hypertension, smoking status, and alcohol consumption.

We also utilized generalized additive models and smooth curve fitting to investigate potential nonlinear associations between AIP and stroke. If a nonlinear relationship was detected, a 2-piecewise linear regression model was applied to determine threshold or saturation effects. Likelihood ratio tests were used to compare the segmented model with a standard linear model to assess the statistical significance of threshold effects.

Subgroup analyses were conducted to further explore the association between AIP and stroke across various strata, including sex (male/female), race/ethnicity (Mexican American, other Hispanic, non-Hispanic White, non-Hispanic Black, other races including multi-racial), education level (less than high school, high school, greater than high school), marital status (married/living with partner, widowed/divorced/separated, never married), BMI categories (<25, 25–30, >30), alcohol consumption (yes/no), smoking status (yes/no), and hypertension status (yes/no). These variables were also treated as potential effect modifiers, and interaction terms were included in the models. Likelihood ratio tests were used to assess heterogeneity in associations across subgroups.

Missing values were imputed using the median for continuous variables and the mode for categorical variables, based on available data. Model adequacy was assessed through standard diagnostic procedures, including goodness-of-fit tests for logistic regression models. To account for multiple testing in subgroup analyses, appropriate statistical corrections were applied when necessary. Sensitivity analyses were conducted to ensure robustness of findings by examining the consistency of results across different analytical approaches. All statistical analyses were conducted using R software (version 4.4.1; R Foundation for Statistical Computing, Vienna, Austria, http://www.r-project.org) and EmpowerStats (version 8.0; EmpowerStats Studio, Beijing, China, http://www.empowerstats.com), with a two-sided *P*-value < .05 considered statistically significant.

## 3. Results

### 3.1. Baseline characteristics of participants

Distribution assessments guided the selection of appropriate statistical tests for all analyses. A total of 16,834 individuals were included in this study, with a mean age of 48.07 ± 16.86 years. Baseline characteristics varied significantly across tertiles of the AIP (all *P* < .01). Participants in the highest AIP tertile (*Q*_3_) tended to be older (49.32 ± 15.82 years) compared to those in the lowest tertile (*Q*_1_: 46.47 ± 17.34 years, *P* < .01). The proportion of males was notably higher in *Q*_3_ (62.09%) than in *Q*_1_ (36.56%; *P* < .01). Ethnic distribution also differed among AIP tertiles: Mexican Americans (10.56%) and non-Hispanic Whites (69.23%) were more prevalent in *Q*_3_, whereas *Q*_1_ had a greater proportion of non-Hispanic Blacks (15.43%; *P* < .01). The overall prevalence of stroke was 3.03%, with stroke frequency increasing alongside AIP tertiles (*Q*_1_: 2.08%; *Q*_2_: 2.94%; *Q*_3_: 4.08%; *P* < .01). Statistically significant differences were observed across the tertiles in various demographic, clinical, and biochemical parameters, including: age, sex, race/ethnicity, education level, marital status, poverty–income ratio, BMI, waist circumference, RBC count, HGB, PLT count, TC, TG, HDL-C, LDL-C, albumin, ALT, AST, alkaline phosphatase, serum creatinine, blood urea nitrogen, glycated HGB, fasting plasma glucose, monocyte count, smoking status, diabetes, hypertension, alcohol consumption, and stroke history (all *P* < .05). Detailed baseline characteristics of participants across AIP tertiles are presented in Table 1.

**Table 1:** Baseline characteristics of the study population by atherogenic index of plasma tertiles.

AIP	All (N = 16,834)	*Q*_1_ (N = 5610)	*Q*_2_ (N = 5612)	*Q*_3_ (N = 5612)	*P*-value
Age (yr, mean ± SD)	48.07 ± 16.86	46.47 ± 17.34	48.48 ± 17.23	49.32 ± 15.8	<.01
Sex (n, %)					<.01
Male	8289 (49.24%)	2051 (36.56%)	2745 (48.92%)	3485 (62.09%)	
Female	8545 (50.76%)	3559 (63.44%)	2867 (51.09%)	2127 (37.91%)	
Race (n, %)					<.01
Mexican American	2633 (15.64%)	348 (6.21%)	495 (8.82%)	593 (10.56%)	
Other Hispanic	1693 (10.06%)	269 (4.80%)	337 (6.01%)	357 (6.36%)	
Non-Hispanic White	7171 (42.60%)	3701 (65.98%)	3794 (67.57%)	3885 (69.23%)	
Non-Hispanic Black	3433 (20.39%)	866 (15.43%)	578 (10.29%)	325 (5.78%)	
Other race-including multi-racial	1904 (11.31%)	426 (7.59%)	411 (7.32%)	453 (8.07%)	
Education level (n, %)					<.01
Below high school	4300 (25.54%)	694 (12.37%)	971 (17.30%)	1154 (20.57%)	
High school	8687 (51.60%)	2862 (51.01%)	3107 (55.33%)	3141 (55.99%)	
Above high school	3847 (22.85%)	2054 (36.62%)	1534 (27.37%)	1317 (23.45%)	
Marital status (n, %)					<.01
Married/living with a partner	10106 (60.03%)	3475 (61.94%)	3587 (63.92%)	3727 (66.41%)	
Widowed/divorced/separated	3773 (22.41%)	1003 (17.87%)	1062 (18.91%)	1078 (19.21%)	
Never married	2955 (17.55%)	1132 (20.19%)	964 (17.18%)	807 (14.38%)	
The ratio of family income-to-poverty, PIR, (mean ± SD)	2.96 ± 1.58	3.10 ± 1.58	2.93 ± 1.58	2.84 ± 1.58	<.01
Waist circumference (cm, mean ± SD)	99.24 ± 16.24	91.64 ± 14.76	99.64 ± 15.05	106.67 ± 15.28	<.01
BMI (kg/m², mean ± SD)	29.01 ± 6.88	26.38 ± 6.20	29.19 ± 6.73	31.54 ± 6.71	<.01
RBC (10^9^/L, mean ± SD)	4.73 ± 0.48	4.61 ± 0.46	4.73 ± 0.48	4.86 ± 0.48	<.01
Hemoglobin (g/dL, mean ± SD)	14.34 ± 1.47	13.97 ± 1.40	14.34 ± 1.46	14.73 ± 1.46	<.01
PLT (10^9^/L, mean ± SD)	244.80 ± 64.53	238.78 ± 61.25	247.17 ± 65.43	248.64 ± 66.47	<.01
HDL-C (mg/dL, mean ± SD)	1.41 ± 0.42	1.76 ± 0.43	1.37 ± 0.26	1.08 ± 0.23	<.01
LDL-C (mg/dL, mean ± SD)	2.94 ± 0.90	2.71 ± 0.80	3.03 ± 0.90	3.10 ± 0.96	<.01
TC (mg/dL, mean ± SD)	192.92 ± 41.19	185.20 ± 36.99	190.99 ± 39.44	202.80 ± 44.86	<.01
TG (mmol/L, mean ± SD)	1.43 ± 1.19	0.70 ± 0.21	1.18 ± 0.27	2.41 ± 1.62	<.01
Albumin (g/dL, mean ± SD)	4.23 ± 0.33	4.25 ± 0.33	4.22 ± 0.34	4.22 ± 0.34	<.01
ALT (U/L, mean ± SD)	25.21 ± 17.74	21.64 ± 17.17	24.29 ± 15.28	29.81 ± 19.51	<.01
AST (U/L, mean ± SD)	25.15 ± 16.62	24.56 ± 19.15	24.44 ± 14.05	26.46 ± 16.10	<.01
ALP (U/L, mean ± SD)	68.35 ± 24.11	64.31 ± 22.64	68.53 ± 21.95	72.35 ± 26.82	<.01
BUN (mg/dL, mean ± SD)	13.58 ± 5.43	13.26 ± 4.92	13.56 ± 5.41	13.91 ± 5.90	<.01
Scr (mg/dL, mean ± SD)	0.89 ± 0.37	0.85 ± 0.36	0.89 ± 0.31	0.92 ± 0.43	<.01
HbA1c (mmol/L, mean ± SD)	5.64 ± 0.95	5.43 ± 0.65	5.60 ± 0.87	5.88 ± 1.20	<.01
FPG (10^9^/L, mean ± SD)	5.94 ± 1.75	5.53 ± 1.15	5.84 ± 1.48	6.45 ± 2.30	<.01
Monocyte number (10^9^/L, mean ± SD)	0.54 ± 0.19	0.51 ± 0.17	0.55 ± 0.18	0.57 ± 0.23	<.01
Hypertensive (n, %)					<.01
Yes	7363 (43.74%)	1647 (29.36%)	2216 (39.49%)	2731 (48.66%)	
No	9471 (56.26%)	3963 (70.64%)	3396 (60.51%)	2881 (51.34%)	
Diabetes (n, %)					<.01
Yes	3465 (20.58%)	466 (8.31%)	808 (14.40%)	1375 (24.50%)	
No	13369 (79.42%)	5144 (91.69%)	4804 (85.61%)	4237 (75.50%)	
Coronary heart disease (n, %)					<.01
Yes	716 (4.25%)	143 (2.55%)	196 (3.50%)	273 (4.86%)	
No	16118 (95.75%)	5467 (97.45%)	5416 (96.50%)	5339 (95.14%)	
Drinking (n, %)					<.01
Yes	9866 (58.61%)	3400 (60.60%)	3516 (62.66%)	3627 (64.66%)	
No	6968 (41.39%)	2210 (39.40%)	2096 (37.34%)	1985 (35.34%)	
Smoking (n, %)					<.01
Yes	7603 (45.16%)	2191 (39.05%)	2523 (44.95%)	3001 (53.45%)	
No	9231 (54.84%)	3419 (60.95%)	3089 (55.05%)	2611 (46.55%)	
Stroke (n, %)					<.01
Yes	673 (4.00%)	144 (2.57%)	166 (2.95%)	200 (3.57%)	
No	16161 (96.00%)	5466 (97.43%)	5446 (97.05%)	5412 (96.43%)	

AIP = atherogenic index of plasma, ALT = alanine aminotransferase, AST = aspartate aminotransferase, BMI = body mass index, BUN = blood urea nitrogen, FPG = fasting plasma glucose, HbA1c = glycated hemoglobin, HDL-C = high-density lipoprotein cholesterol, LDL-C = low-density lipoprotein cholesterol, PIR = poverty–income ratio, PLT = platelet, RBC = red blood cell, Scr = serum creatinine, SD = standard deviation, TC = total cholesterol, TG = triglycerides.

### 3.2. Univariate analysis

In the univariate analysis, an increase in the AIP was significantly associated with a higher likelihood of stroke. Specifically, each 1-unit increase in AIP was associated with a 39% increase in the odds of stroke (odds ratio [OR] = 1.39, 95% confidence interval [CI]: 1.11–1.74, *P* = .0042). Several clinical and biochemical factors were also found to be positively associated with stroke, including age, BMI, waist circumference, TG levels, monocyte count, PLT count, glycated HGB, fasting plasma glucose, AST, alkaline phosphatase, serum creatinine, blood urea nitrogen, hypertension, diabetes, coronary heart disease, and smoking status. Conversely, higher values of poverty–income ratio, HDL-C, LDL-C, TC, RBC count, HGB, albumin, ALT, and alcohol consumption were negatively correlated with the occurrence of stroke (Table 2).

**Table 2:** Univariate analysis.

Variable	Statistics	OR (95% CI), *P*-value
AIP	−0.05 ± 0.33	1.39 (1.11, 1.74) <.01
Age (yr, mean ± SD)	50.28 ± 17.71	1.07 (1.06, 1.07) <.01
Sex (n, %)		
Male	8289 (49.24%)	0.95 (0.81, 1.11) .51
Female	8545 (50.76%)	1.0
Race (n, %)		
Mexican American	2633 (15.64%)	1.0
Other Hispanic	1693 (10.06%)	1.13 (0.76, 1.69) .55
Non-Hispanic White	7171 (42.60%)	2.22 (1.68, 2.95) <.01
Non-Hispanic Black	3433 (20.39%)	2.60 (1.93, 3.51) <.01
Other race-including multi-racial	1904 (11.31%)	0.98 (0.65, 1.46) .91
Education level (n, %)		
Below high school	4300 (25.54%)	1.0
High school	8687 (51.60%)	0.67 (0.57, 0.79) <.01
Above high school	3847 (22.85%)	0.36 (0.28, 0.46) <.01
Marital status (n, %)		
Married/living with a partner	10106 (60.03%)	1.0
Widowed/divorced/separated	3773 (22.41%)	2.04 (1.73, 2.41) <.01
Never married	2955 (17.55%)	0.54 (0.41, 0.72) <.01
The ratio of family income-to-poverty, PIR, (mean ± SD)	2.50 ± 1.54	0.82 (0.78, 0.87) <.01
BMI (kg/m², mean ± SD)	29.12 ± 6.92	1.02 (1.01, 1.03) <.01
Waist circumference (cm, mean ± SD)	99.28 ± 15.93	1.02 (1.01, 1.02) <.01
HDL-C (mg/dL, mean ± SD)	1.39 ± 0.42	0.88 (0.73, 1.07) .20
TG (mg/dL, mean ± SD)	1.43 ± 1.25	1.03 (0.98, 1.09) .21
LDL-C (mg/dL, mean ± SD)	2.93 ± 0.91	0.73 (0.67, 0.80) <.01
TC (mg/dL, mean ± SD)	192.01 ± 41.69	0.99 (0.99, 1.00) <.01
Monocyte number (10^9^/L, mean ± SD)	0.53 ± 0.20	2.58 (1.86, 3.58) <.01
RBC (10^9^/L, mean ± SD)	4.70 ± 0.51	0.46 (0.40, 0.54) <.01
Hemoglobin (g/dL, mean ± SD)	14.15 ± 1.55	0.81 (0.77, 0.85) <.01
PLT (10^9^/L, mean ± SD)	244.27 ± 66.70	1.00 (1.00, 1.00) <.01
HbA1c (mmol/L, mean ± SD)	5.78 ± 1.11	1.25 (1.20, 1.31) <.01
FPG (10^9^/L, mean ± SD)	6.11 ± 2.03	1.11 (1.09, 1.14) <.01
Albumin (g/dL, mean ± SD)	4.20 ± 0.34	0.27 (0.22, 0.33) <.01
ALT (U/L, mean ± SD)	25.08 ± 19.15	0.99 (0.98, 0.99) <.01
AST (U/L, mean ± SD)	25.44 ± 19.45	1.00 (0.99, 1.00) .34
ALP (U/L, mean ± SD)	70.58 ± 25.13	1.01 (1.01, 1.01) <.01
BUN (mg/dL, mean ± SD)	13.70 ± 6.02	1.07 (1.06, 1.08) <.01
Scr (mg/dL, mean ± SD)	0.90 ± 0.46	1.60 (1.45, 1.76) <.01
Drinking, n (%)		
Yes	9866 (58.61%)	0.80 (0.68, 0.93) <.01
No	6968 (41.39%)	1.0
Hypertensive (n, %)		
Yes	7363 (43.74%)	5.57 (4.60, 6.76) <.01
No	9471 (56.26%)	1.0
Diabetes (n, %)		
Yes	3465 (20.58%)	3.12 (2.66, 3.65) <.01
No	13369 (79.42%)	1.0
Coronary heart disease (n, %)		
Yes	716 (4.25%)	5.73 (4.63, 7.10) <.01
No	16118 (95.75%)	1.0
Smoking (n, %)		
Yes	7603 (45.16%)	1.88 (1.61, 2.20) <.01
No	9231 (54.84%)	1.0

AIP = atherogenic index of plasma, ALP = alkaline phosphatase, ALT = alanine aminotransferase, AST = aspartate aminotransferase, BMI = body mass index, BUN = blood urea nitrogen, CI = confidence interval, FPG = fasting plasma glucose, HbA1c = glycated hemoglobin, HDL-C = high-density lipoprotein cholesterol, LDL-C = low-density lipoprotein cholesterol, OR = odds ratio, PIR = poverty–income ratio, PLT = platelet, RBC = red blood cell, Scr = serum creatinine, SD = standard deviation, TC = total cholesterol, TG = triglycerides.

### 3.3. Association between AIP and stroke

The association between the AIP and stroke is presented in Table 3. Our analysis revealed a consistent positive correlation between AIP and the risk of stroke across all models. In the fully adjusted model (model III), each 1-unit increase in AIP was associated with a 47% increase in the odds of experiencing a stroke (OR = 1.47, 95% CI: 1.12–1.93). Furthermore, when AIP was analyzed as a categorical variable by tertiles, the trend remained significant. In model III, individuals in the highest AIP tertile had a 32% higher risk of stroke compared to those in the lowest tertile (OR = 1.32, 95% CI: 1.07–1.63, *P* < .01).

**Table 3:** The association between AIP and stroke.

Variable	Non-adjusted OR (95% CI), *P*-value	Adjust l OR (95% CI), *P*-value	Adjust ll OR (95% Cl), *P*-value
AIP 1	1.39 (1.11, 1.74) <.0	1.54 (1.19, 1.99) <.01	1.47 (1.12, 1.93) <.01
Trisections of AIP			
*Q*_1_	1.0 (Reference)	1.0 (Reference)	1.0 (Reference)
*Q*_2_	1.13 (0.93, 1.37) .23	1.04 (0.84, 1.27) .73	1.02 (0.82, 1.26) .85
*Q*_3_	1.36 (1.13, 1.65) <.01	1.37 (1.12, 1.68) <.01	1.32 (1.07, 1.63) <.01
*P* for trend	<.01	<.01	.02

In the sensitivity analysis, AIP was converted from a continuous variable to a categorical variable (tertiles).

Model 1: No covariates were adjusted for.

Model 2: Adjusted for gender, age, race, education level, marital status, and family income-to-poverty ratio.

Model 3: Adjusted for gender, age, race, education level, marital status, ratio of family income-to-poverty, LDL-C, TC, ALT, AST, HGB concentration, RBC count, PLT count, monocyte count, hypertension, smoking, and drinking status.

AIP = atherogenic index of plasma, ALT = alanine aminotransferase, AST = aspartate aminotransferase, CI = confidence interval, HGB = hemoglobin, LDL-C = low-density lipoprotein cholesterol, OR = odds ratio, PLT = platelet, *Q*_1_ = tertile 1, *Q*_2_ = tertile 2, *Q*_3_ = tertile 3, RBC = red blood cell, TC = total cholesterol.

**Figure 2.**
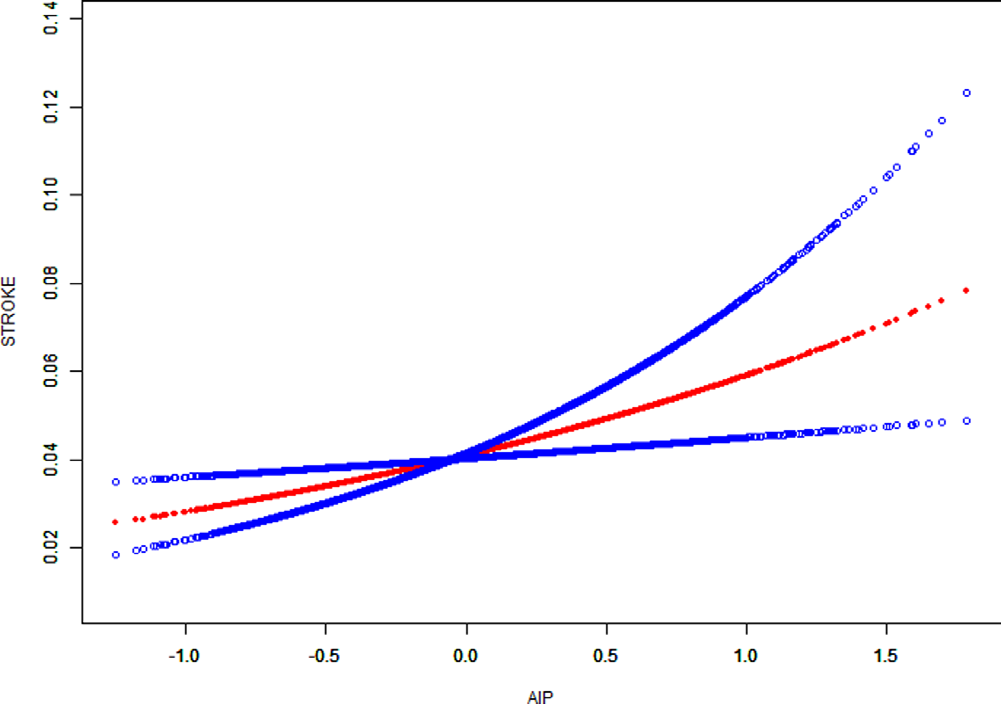
The smooth curve fitting of AIP and stroke. A linear relationship between AIP and stroke was detected using the generalized additive model (GAM). Adjustments were made for gender, age, race, education level, marital status, ratio of family income-to-poverty, LDL-C, TC, ALT, AST, HGB concentration, RBC count, PLT count, monocyte count, hypertension, smoking, and drinking status. AIP = atherogenic index of plasma, ALT = alanine aminotransferase, AST = aspartate aminotransferase, GAM = generalized additive model, HGB = hemoglobin, LDL-C = low-density lipoprotein cholesterol index, PLT = platelet, RBC = red blood cell, TC = total cholesterol.

These findings were further supported by smooth curve fitting analysis, which visually demonstrated a positive linear relationship between AIP and the incidence of stroke (Fig. 2). Diagnostic assessments confirmed adequate model performance across all regression models. A linear relationship between AIP and stroke was detected using the generalized additive model.

### 3.4. Subgroup analysis

To evaluate the stability of the association between AIP and stroke across different population subgroups, we conducted stratified analyses and interaction tests based on sex, race/ethnicity, educational attainment, marital status, alcohol consumption, hypertension, and smoking status. As shown in Table 4, the positive relationship between AIP and stroke remained consistent across all examined subgroups. No significant interactions were observed for any of the stratification variables (all interaction *P*-values > .05), indicating that the association between AIP and stroke was not modified by sex, race, education level, marital status, alcohol intake, hypertension, or smoking status. These results suggest that the observed relationship between AIP and stroke risk may be broadly applicable across diverse demographic and clinical populations. Statistical corrections for multiple comparisons were applied to subgroup analyses, and all main findings remained robust.

**Table 4:** Subgroup analysis and interaction test of AIP and stroke.

Subgroup	OR (95% CI), *P*-value	*P* for interaction
Sex		.89
Female	1.33 (0.90, 1.97) .16	
Male	1.43 (0.97, 2.11) .07	
Race		.14
Mexican American	1.18 (0.43, 3.20) .75	
Other Hispanic	1.68 (0.45, 6.31) .44	
Non-Hispanic White	1.35 (0.91, 2.00) .13	
Non-Hispanic Black	1.78 (1.06, 2.99) .03	
Other race-including	0.35 (0.10, 1.21) .10	
Education level		.63
Below high school	1.19 (0.75, 1.90) .45	
High school	1.59 (1.07, 2.34) .02	
Above high school	1.39 (0.62, 3.09) .42	
Marital status		.44
Married/living with partner	1.34 (0.91, 1.97) .15	
Widowed/divorced/separated	1.31 (0.85, 2.04) .22	
Never married	2.50 (1.01, 6.19) .04	
Drinking		.62
Yes	1.42 (0.99, 2.03) .06	
No	1.28 (0.83, 1.96) .26	
Hypertensive		.91
Yes	1.34 (0.99, 1.83) .06	
No	1.44 (0.78, 2.65) .24	
Smoking		.56
Yes	1.42 (1.00, 2.01) .05	
No	1.32 (0.85, 2.05) .22	

Adjusted for gender, age, race, education level, marital status, ratio of family income-to-poverty, LDL-C, TC, ALT, AST, HGB concentration, RBC count, PLT count, monocyte count, hypertension, smoking, and drinking status.

AIP = atherogenic index of plasma, ALT = alanine aminotransferase, AST = aspartate aminotransferase, CI = confidence interval, HGB = hemoglobin, LDL-C = low-density lipoprotein cholesterol, OR = odds ratio, PLT = platelet, RBC = red blood cell, TC = total cholesterol.

Sensitivity analyses confirmed the consistency of our primary findings across different analytical specifications, supporting the robustness of the observed association between AIP and stroke risk.

## 4. Discussion

This cross-sectional study involving 23,389 participants aimed to investigate the potential association between AIP and stroke incidence. We found a positive correlation between AIP and stroke prevalence, meaning individuals with higher AIP levels are more likely to experience a stroke. Subgroup analyses and interaction tests indicated that factors such as gender, age, coronary artery disease, hypertension, or diabetes did not significantly affect this association, confirming the robustness of the positive relationship across various demographic backgrounds. These findings suggest that elevated AIP could be an independent risk factor for stroke, underscoring the importance of AIP in the prevention and management of stroke.

To the best of our knowledge, this is the first cross-sectional study assessing the association between AIP and stroke. In this study, our analysis unexpectedly found a negative association between LDL-C, RBC, and stroke risk, which appears to be inconsistent with existing epidemiological evidence. This discrepancy may be due to reverse causality, where lower LDL-C levels reflect a chronic disease state rather than a protective effect against stroke. Similarly, a reduced RBC count may indicate underlying inflammation or anemia, which could impact cerebrovascular health. Additionally, the observed protective effect of alcohol consumption may be influenced by the “healthy drinker” effect, where moderate drinkers tend to have better overall health compared to former drinkers. Subsequently, we included multiple covariates in our analysis to minimize potential confounding effects on the association between AIP and stroke. LDL-C and TC are important risk factors for atherosclerosis, and previous studies have demonstrated their strong correlation with stroke risk.^[17]^ Hematological parameters (such as HGB, white blood cells, RBC, and PLT) reflect the body’s inflammatory status, hemodynamic changes, and coagulation function, all of which may influence stroke occurrence.^[18]^ Additionally, socioeconomic and lifestyle factors, including gender, age, race, education level, income level, hypertension, smoking, and alcohol consumption, were incorporated to control for potential confounding effects. The study focuses on the plasma AIP, which is calculated by performing a logarithmic transformation of the TG to HDL-C ratio. TG levels reflect the lipid metabolic status in the body, while HDL-C is associated with anti-atherosclerotic properties. AIP offers a more comprehensive representation of the lipid metabolic balance and its relationship with atherosclerotic risk. Increasing evidence indicates that AIP is associated with the occurrence and progression of many diseases. Zheng et al reported that long-term exposure to high AIP levels may increase the risk of ischemic stroke.^[19]^ Ding et al found that TG-rich lipoproteins are a new target for atherosclerosis.^[20]^ Qu et al suggested that baseline high AIP levels in individuals with deglycation might indicate a subgroup at higher risk for stroke, although AIP levels in middle-aged and older adults without deglycation did not affect stroke incidence. Meanwhile, you et al reported no significant association between high AIP levels and overall or cardiovascular mortality risk, but among women aged over 65, diabetes-related mortality risk increased progressively with rising AIP levels.^[21]^ These results suggest that higher AIP, compared to lower AIP, may be associated with worse outcomes.

Although direct studies linking HGB/red blood cell distribution width ratio to stroke are relatively rare, studies have shown that AIP can serve as an independent predictor of prognosis in patients with type 2 diabetes.^[22]^ Hyperglycemia, insulin resistance, and hyperinsulinemia can lead to lipid metabolic disorders, oxidative stress, and vascular endothelial injury, ultimately worsening coronary atherosclerosis.^[23]^ A meta-analysis of 15 case-control studies indicated that AIP predicts diabetes risk better than other lipid components.^[24]^ Chronic inflammation may also play an important role in the association between AIP and cardiovascular disease-specific mortality. High TG levels promote the formation of small dense low-density lipoprotein particles, which are more likely to penetrate the vascular endothelium, triggering inflammatory responses and promoting atherosclerosis. Additionally, TG breakdown products, such as free fatty acids, can activate the nuclear factor κB signaling pathway, inducing the release of pro-inflammatory factors (such as tumor necrosis factor-α, interleukin-6), and driving systemic inflammation. HDL-C has anti-atherosclerotic functions, including cholesterol reverse transport, antioxidant, and anti-inflammatory effects. A decrease in HDL-C levels weakens these protective mechanisms, making the vascular wall more vulnerable to damage, thereby exacerbating lipid metabolic disorders due to chronic inflammation. Given that AIP is calculated from the serum TC and HDL-C ratio, the use of anti-hyperlipidemic drugs may impact overall vascular and lipid metabolic levels, and further exploration of AIP’s role in all-cause mortality and cardiovascular disease-specific mortality in hypertensive patients with varying medication use is warranted.

This study found a positive association between the AIP and stroke risk; however, several limitations need to be addressed. Our study is based on the NHANES database, which provides extensive health and disease-related information but does not distinguish specific stroke subtypes (e.g., ischemic stroke and hemorrhagic stroke). Given the significant differences in pathophysiological mechanisms, risk factors, and prognosis between these subtypes, this limitation may affect our ability to explore the relationship between AIP and specific stroke subtypes in depth. Future studies should employ prospective cohort designs or integrate other databases to further analyze the role of AIP in different stroke subtypes.

The strength of this study lies in its use of NHANES data, which are collected using a stratified, multistage probability sampling strategy, enhancing the reliability and representativeness of the findings. The nationwide dataset, which accounts for sample weights, allows for generalization to the broader US population. Regression analyses adjusted for covariates and the large sample size enabled subgroup analyses to confirm the association between AIP and stroke across different population contexts. However, the study does have several limitations. First, stroke diagnosis was based on self-reported medical history, with no detailed information on stroke subtype or staging. Reliance on self-reporting may introduce recall bias, particularly regarding stroke diagnosis, potentially affecting the accuracy of symptom and severity reporting and, thus, the study’s reliability. Second, while adjustments were made for several potential covariates, other confounding factors, such as medication use, certain comorbidities (e.g., brain tumors, brain injury, atrial fibrillation), and social/environmental factors, were not recorded in NHANES and could not be fully accounted for. Additionally, the study’s findings are based on a single country and ethnic background, so the generalizability to other ethnicities or countries remains to be verified. Finally, due to the cross-sectional design, we cannot establish a causal relationship between AIP and stroke risk. Future randomized controlled trials would be helpful to validate this association and explore its underlying mechanisms.

## 5. Conclusion

This study demonstrates a positive correlation between AIP and the likelihood of stroke, with individuals having higher AIP levels being at a greater risk of stroke. These findings highlight the importance of AIP in identifying patients at risk for stroke. However, this study also has some limitations, and further research is needed to validate our findings.

## Acknowledgments

We would like to thank all participants in NHANES study.

## Author contributions

**Conceptualization:** Ziyi Liang, Huasheng Zhang, Yang Xiong.

**Data curation:** Ziyi Liang, Huasheng Zhang, Yang Xiong, Xiaohu Zhang.

**Formal analysis:** Ziyi Liang, Huasheng Zhang, Yuliang Chen, Peilong Li.

**Investigation:** Yang Xiong, Xiaohu Zhang.

**Methodology:** Yuliang Chen, Peilong Li.

**Project administration:** Jun Liang.

**Supervision:** Jun Liang.

**Validation:** Qiyou Yi, Kangkang Xia, Jun Liang.

**Writing – original draft:** Ziyi Liang, Huasheng Zhang, Yang Xiong.

**Writing – review & editing:** Jun Liang.
